# Quad-Band Terahertz Metamaterial Sensor Based on Dual Near-Perfect Absorption for Fingerprint Identification of Cancer Cells

**DOI:** 10.3390/s26216807

**Published:** 2026-10-28

**Authors:** Aykut Coskun, Ahmet Ozmen

**Affiliations:** 1 Department of Electrical and Electronics Engineering, Engineering Faculty, Bayburt University, 69000 Bayburt, Turkey; 2 Department of Electronics and Communication Engineering, Of Technology Faculty, Karadeniz Technical University, 61830 Trabzon, Turkey; ahmetozmen@ktu.edu.tr

**Keywords:** terahertz, metamaterial sensor, quad-band absorber, cancer identification, biosensor

## Abstract

Early and accurate cancer diagnosis remains a critical challenge in clinical oncology due to the limitations of traditional, destructive labeling methods. To address this gap, this study proposes a novel, label-free quad-band terahertz (THz) metamaterial biosensor designed for high-precision fingerprint identification of cancer cell lines. Utilizing a classic tri-layer sandwich architecture with a top patterned geometry of dual rings, numerical simulations were systematically performed via CST Studio Suite 2024 to evaluate the sensor’s electromagnetic performance. The proposed sensor established four distinct resonance channels at 2.274, 2.349, 2.552, and 2.615 THz, achieving ultra-deep absorption profiles down to −40.36 dB. When numerically investigated against six distinct biological lineages (Basal, HeLa, Jurkat, MCF-7, MDA-MB-231, and PC-12), the sensor successfully converted minor intracellular refractive index fluctuations into highly resolved, non-overlapping spectral displacements. Performance evaluations revealed an exceptional maximum optical sensitivity of 9642.8 GHz/RIU alongside a monumental Figure of Merit (FOM) of 1928.56 RIU^−1^ for breast cancer cells (MCF-7), while demonstrating a peak Quality factor (*Q*-factor) of 870.33 for leukemia cells (Jurkat) and a high sensitivity of 6600.0 GHz/RIU for skin cancer cells (Basal). Ultimately, these decoupled multi-channel fingerprint outputs provide strong diagnostic redundancy, confirming the designed metamaterial absorber as a highly promising tool for next-generation, non-invasive early cancer diagnostic applications.

## 1. Introduction

Cancer remains one of the leading causes of mortality worldwide, necessitating the development of rapid, highly accurate, and non-invasive diagnostic tools [1,2,3,4,5,6,7,8,9,10,11]. Traditional diagnostic modalities, including histopathology and polymerase chain reactions, often suffer from being destructive, time-consuming, and require complex labeling processes [12,13,14,15,16,17,18,19]. To overcome these limitations, terahertz (THz) radiation has emerged as a revolutionary alternative in biomedical imaging and sensing due to its non-ionizing nature and its low photon energy, which prevents damage to biological tissues [20,21,22,23,24,25,26]. Crucially, the vibrational and rotational energy levels of biomolecules fall precisely within the THz gap, allowing for the label-free detection of unique spectral “fingerprints” of biological samples, including different types of cancer cells [27,28,29,30,31].

Despite its immense potential, raw biological materials exhibit low interaction cross-sections with THz waves due to the mismatch between the subwavelength dimensions of cells and the relatively long THz wavelengths [32,33]. To drastically amplify these light-matter interactions, metamaterials (MMs)—engineered artificial structures with extraordinary electromagnetic properties—have been widely adopted [34,35]. Specifically, metamaterial perfect absorbers (MPAs) are employed in refractive index biosensing because they trap incident waves to generate near-unity absorption peaks [36,37]. When a target analyte, such as a cancer cell, is placed on the surface of the sensor, it alters the ambient dielectric environment, causing measurable shifts in the resonance frequencies [38,39,40].

To address these critical demands, this paper proposes a novel quad-band terahertz metamaterial sensor based on near-perfect absorption for the high-precision fingerprint identification of cancer cells. The proposed sensor utilizes a meticulously engineered geometric unit cell that yields four distinct, narrow-band absorption peaks, each achieving near-unity absorption efficiency. By leveraging the strong localized electromagnetic field enhancement at these four distinct frequencies, the sensor dramatically amplifies the weak light-matter interactions inherent to subwavelength biological samples.

Through rigorous numerical simulations, we investigate and computationally assess the sensor’s performance against the aforementioned cancer cell lines by monitoring the discrete frequency shifts induced by their specific refractive index boundaries. The calculated performance metrics, including sensitivity (*S*) and Figure of Merit (FOM), demonstrate that the proposed design offers superior multi-spectral resolution, making it a promising candidate for next-generation, label-free biomedical diagnostics.

## 2. Structure Design and Geometric Configuration

### 2.1. Parametric Sensitivity and Structural Optimization

**Figure 1:**
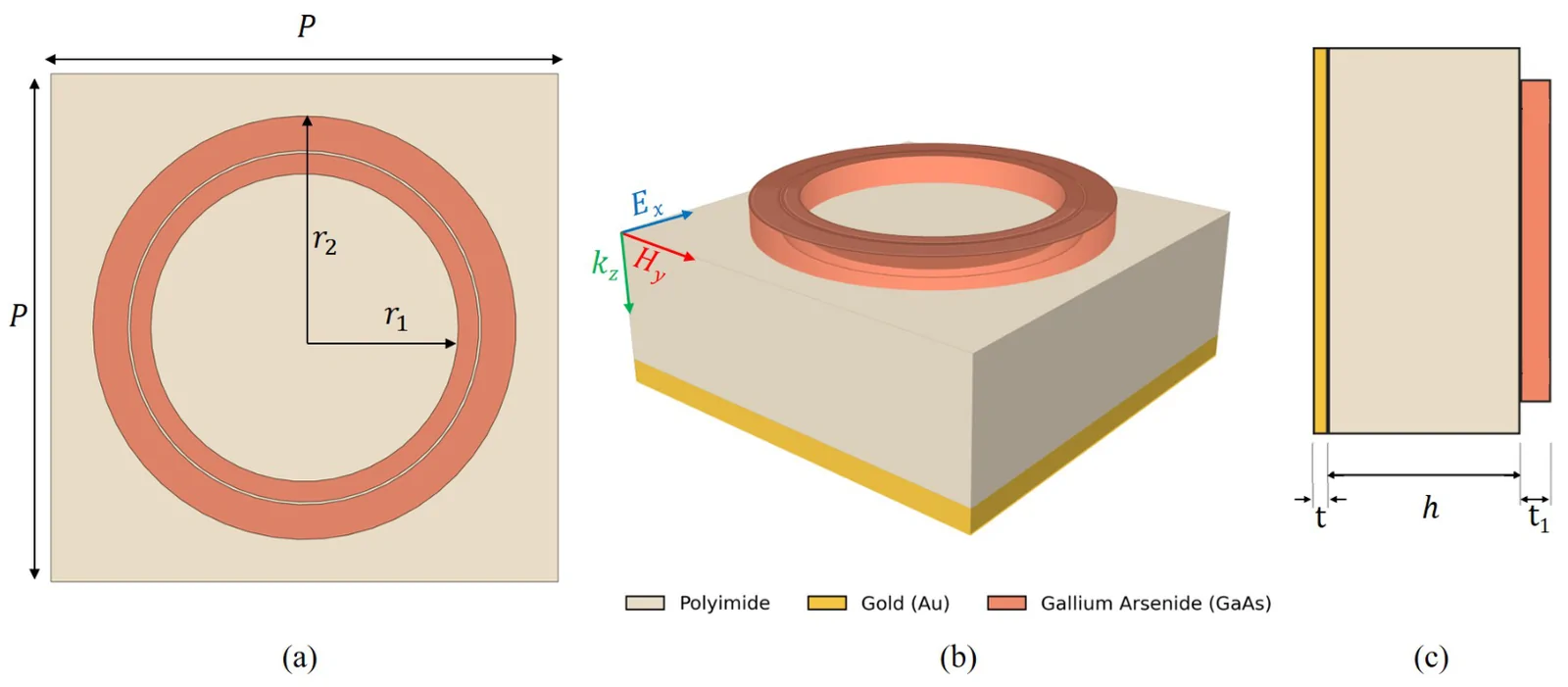
Unit cell geometry of the proposed quad-band terahertz metamaterial sensor: (**a**) top view, (**b**) 3D perspective view, and (**c**) side cross-sectional view.

The unit cell schematic and geometric parameters of the proposed quad-band terahertz metamaterial sensor are depicted in Figure 1. This specific combination of GaAs rings and underlying layers provides strong electromagnetic field confinement in the terahertz regime. The concentric circular GaAs rings are supported by a polyimide dielectric substrate with a relative dielectric constant ($\varepsilon_{r}$) of 3.5 and a relative permeability ($\mu_{r}$) of 1. The thickness (*h*) of the polyimide substrate material is 52.3 μm. The relative permittivity of 12.96 and a loss tangent of 0.06 are used for GaAs. The present study modeled gold as a lossy medium, with a frequency-independent electrical conductivity of 4.56 $\times \;10^{7}\;S/m$ and relative permeability of $\mu_{r}=1$. To ensure zero transmission (i.e., T(ω)=0), the gold (Au) ground plane with a thickness of t=0.2 μm is designed to be significantly greater than the skin depth.

This structure extends periodically along the *x*- and *y*-axes with a periodicity of L=140.3 μm. Numerical simulations and analyses are performed using CST Microwave Studio, which employs the finite integration technique with a tetrahedral mesh layout. To model an infinitely periodic array, open boundary conditions are enforced along the *z*-axis, whereas periodic boundary conditions are applied to the *x*- and *y*-axes. In the numerical simulations, the cancer cell analyte is modeled as a bulk medium placed on the metasurface structure.

**Figure 2:**
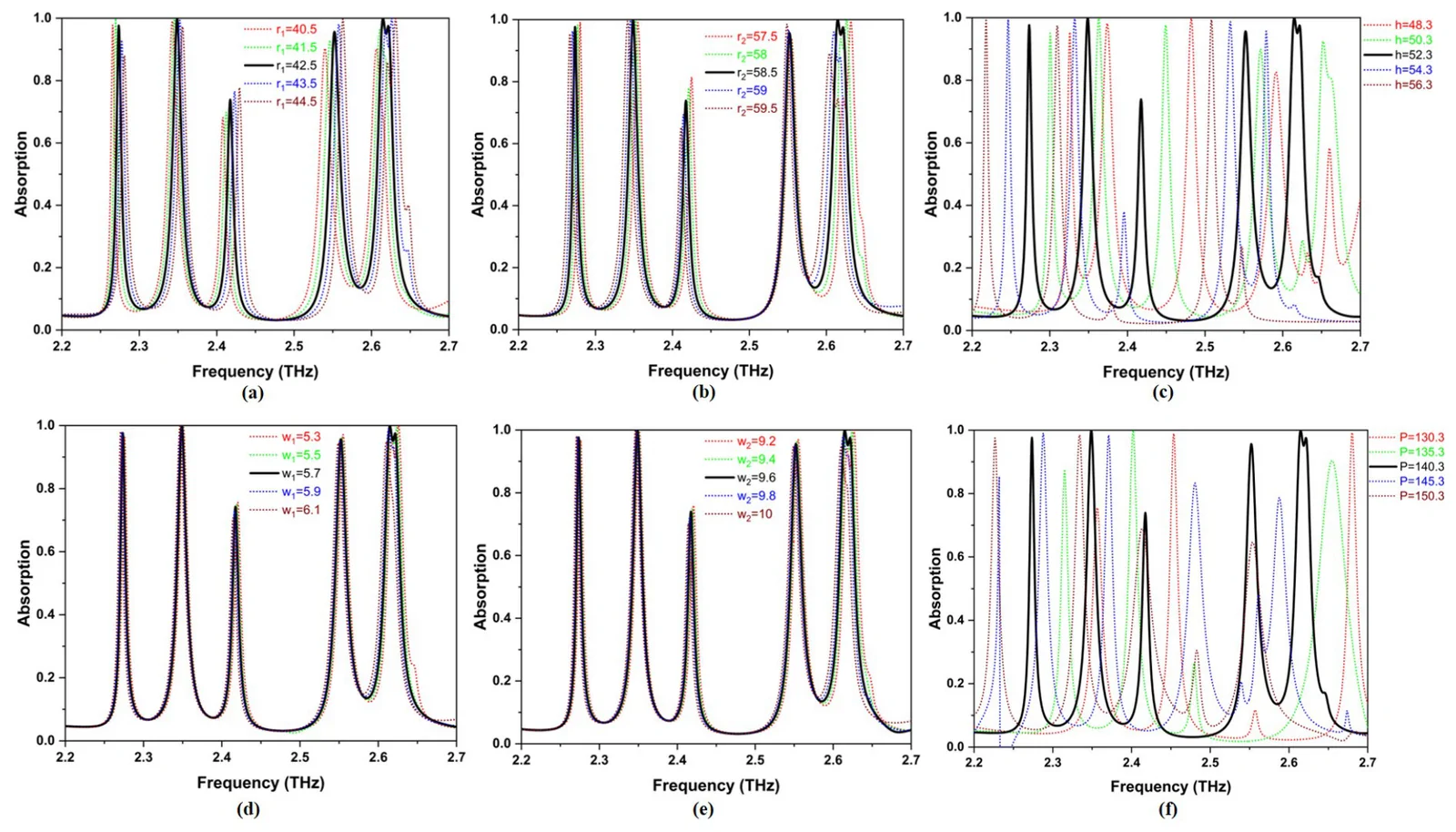
Parametric optimization sweep charts of the proposed terahertz metamaterial sensor as a function of frequency under individual variations of: (**a**) inner radius $r_{1}$, (**b**) outer radius $r_{2}$, (**c**) polyimide substrate thickness *h*, (**d**) inner ring width $w_{1}$, (**e**) outer ring width $w_{2}$, and (**f**) cell periodicity *P*.

To rigorously clarify the design rationale and justify the structural dimensions adopted in the proposed biosensor, a comprehensive parametric scanning analysis was carried out via CST Studio Suite. Figure 2a–f systematically illustrates the dependency of the quad-band absorption performance on the variations of the inner radius ($r_{1}$), outer radius ($r_{2}$), polyimide substrate thickness (*h*), inner width ($w_{1}$), outer width ($w_{2}$), and the periodicity (*P*).

As depicted in Figure 2a,b, modifying the concentric radii ($r_{1}$ and $r_{2}$) directly shifts the central frequencies of the resonance channels, verifying that the physical origins of the multi-band spectra are strongly coupled to the geometric loop sizes. Conversely, the scanning profiles of the ring widths ($w_{1}$ and $w_{2}$) in Figure 2d,e exhibit a more stable, narrow tuning margin, confirming that minor fabrication tolerances in ring widths will not heavily disrupt the target bands.

The sensor architecture fundamentally consists of three distinct layers. The uppermost layer consists of two concentric circular rings, forming a single layer of Gallium Arsenide (GaAs). The thickness ($t_{1}$) of the circular rings is 10 μm. The first inner radius is $r_{1}=42.5$ μm and its width is $w_{1}=5.7$ μm, whereas the second outer radius is $r_{2}=58.5$ μm and its width is $w_{2}=9.6$ μm. The distance between the concentric circular rings is 0.7 μm.

### 2.2. Experimental Fabrication Pathway and THz Characterization Optical Setup

**Figure 3:**
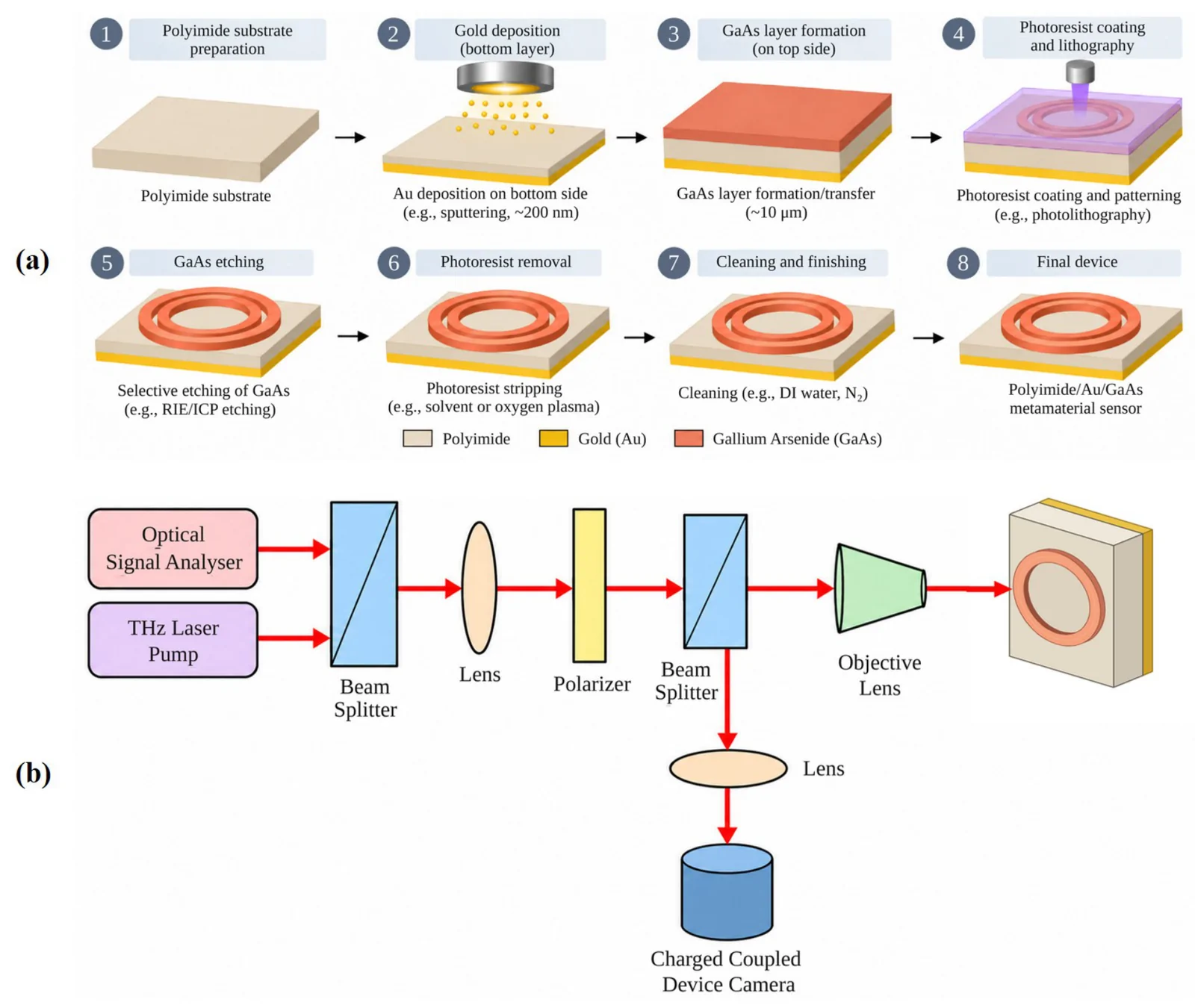
Comprehensive experimental feasibility infrastructure of the proposed quad-band biosensor: (**a**) Schematic flow chart detailing the 8-step microfabrication layer processing, and (**b**) block diagram of the proposed optical terahertz laser pump-probe characterization setup loop.

To validate the practical viability and engineering feasibility of the proposed quad-band metamaterial sensor, a rigorous step-by-step fabrication flow and a complete optical characterization setup have been systematically introduced, as comprehensively illustrated in Figure 3.

The multi-layer manufacturing protocol detailed in Figure 3a is divided into eight primary operational phases: (1) preparation and cleaning of the flexible polyimide dielectric substrate matrix; (2) bottom-side gold (Au) thin-film deposition (∼200 nm) executed via high-precision sputtering or electron-beam evaporation; (3) wafer-scale layer transfer or epitaxial formation of the high-purity, semi-insulating Gallium Arsenide (GaAs) film (∼10 μm) directly onto the top side of the cured polyimide layer; (4) protective photoresist coating and subsequent ultraviolet (UV) photolithography exposure to precisely define the masked concentric ring coordinates; (5) anisotropic selective etching of the unmasked GaAs domains performed via Reactive Ion Etching or Inductively Coupled Plasma (RIE/ICP) systems to guarantee sharp vertical profiles; (6) chemical photoresist stripping and stripping cleanup using solvent or oxygen plasma channels; (7) deionized (DI) water rinse followed by pure nitrogen (N_2_) gas drying; and (8) final deployment of the multi-layered Polyimide/Au/GaAs metamaterial sensor chip.

Concurrently, Figure 3b outlines the strategic optical pump-probe configuration proposed for the experimental monitoring of the bio-sensing channels. The measurement system coordinates a Terahertz Laser Pump source and an Optical Signal Analyser looped through primary beam splitters, alignment lenses, and high-extinction polarizers to strike the metamaterial sensor under normal incidence. The reflected or transmitted spectral responses are subsequently routed through an objective lens matrix and projected onto a high-speed Charged Coupled Device (CCD) Camera system for real-time tracking of the discrete resonance frequency displacements driven by the cancer cell lines.

## 3. Results and Discussion

### 3.1. Evolutionary Design and Resonance Origin

**Figure 4:**
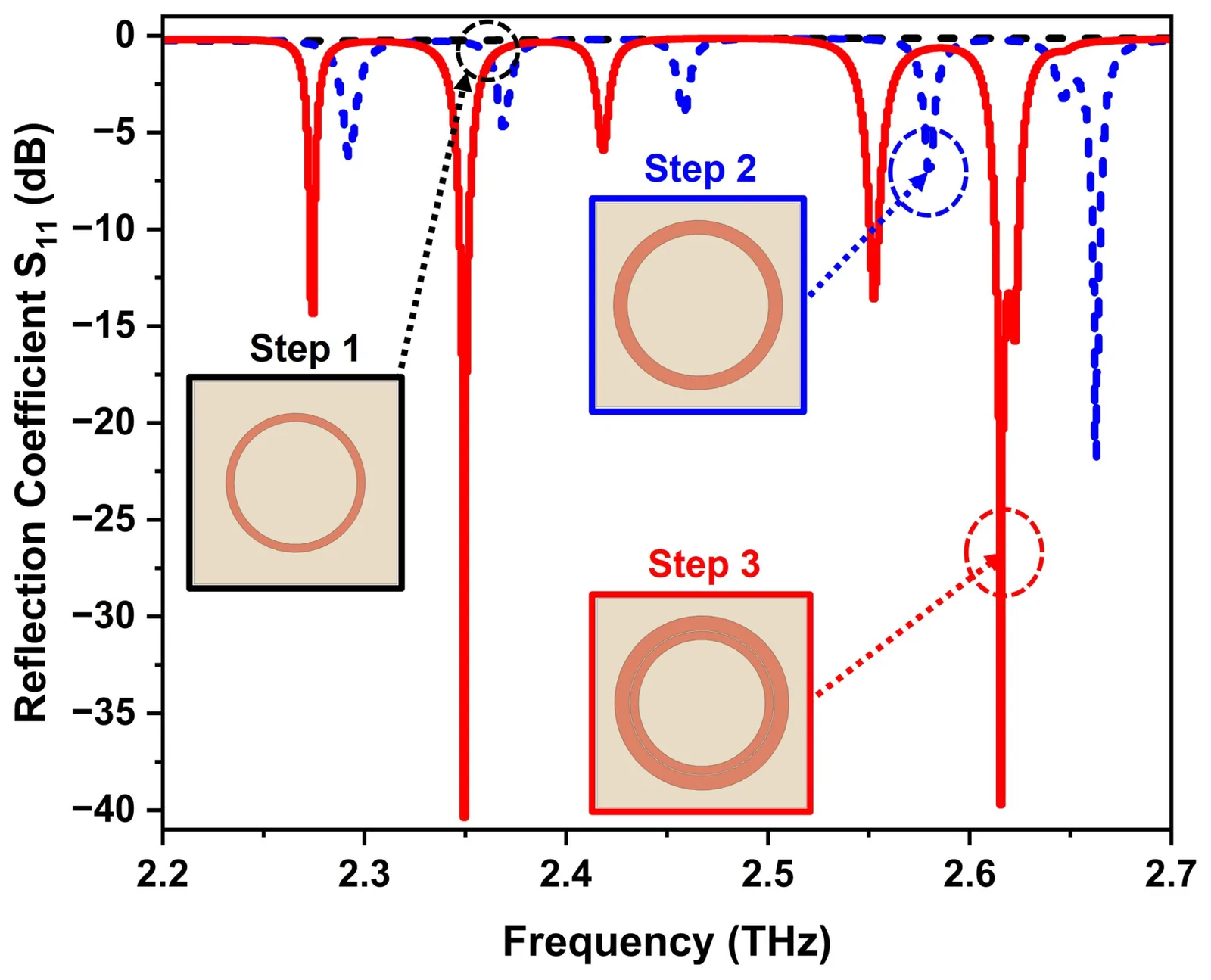
Evolutionary structural analysis of the proposed quad-band terahertz metamaterial sensor plotted as a function of the operational frequency (THz) on the x-axis against the reflection coefficient ($S_{11}$, dB) on the y-axis, highlighting the tracking performance from Step 1 to Step 3.

The numerical simulation of the proposed quad-band metamaterial absorber configuration consists of exactly 14,549 tetrahedral mesh cells and evaluates 1001 individual frequency samples to guarantee maximum computation fidelity. To systematically optimize the geometric layout and resolve the true physical origin of the resonance behaviors, an evolutionary step-by-step layout tracking study was executed by monitoring the reflection coefficient ($S_{11}$, dB) relative function plotted against the operational frequency (THz), as detailed in Figure 4. In the initial design phase, designated as Step 1 (black inset curve in Figure 4), a single inner circular GaAs resonator ring structure alone was simulated, yielding a localized initial reflection dip near 2.27 THz. To enhance the multi-spectral response and improve resonance selectivity, Step 2 introduces the outer concentric GaAs ring topology alone (blue inset curve), inducing alternative localized resonance bands. Finally, in Step 3 (red inset geometry and solid red line), all optimized concentric resonator components are integrated simultaneously. Owing to the strong capacitive-inductive (LC) coupling and local field perturbations taking place between these concentric features, the overall electromagnetic response achieves near-perfect impedance balancing. Consequently, a highly distinct, high-*Q* quad-band resonance characteristic with deep reflection dips is successfully attained at the targeted design center frequencies of 2.274 THz, 2.349 THz, 2.552 THz, and 2.615 THz.

To maintain maximum presentation clarity and prevent severe graphical overlapping, the extensive multi-spectral data generated by tracking the 6 distinct human cancer cell lines across both healthy and malignant states (totaling 12 detailed biological profiles) over 4 independent resonant peaks are systematically consolidated within the performance matrix of Table 1. Rather than deploying an excessive number of individual frequency-dependent absorption curves (which would introduce at least 12 to 24 separate spectral lines, causing visual clutter and degrading readability), this comprehensive tabular layout mathematically organizes the intermediate calculations. By compiling the exact initial resonance nodes, the full width at half maximum (FWHM) values, and the discrete frequency displacements (Δf), the data matrix explicitly demonstrates the sensor’s efficiency metrics.

It should be noted, however, that a direct performance comparison between different terahertz metamaterial sensors presents inherent limitations due to the discrepancies in numerical modeling configurations reported in the literature. Variations in baseline boundary conditions, including analyte layer thickness, implemented refractive index ranges, definition profiles of the figure of merit (FOM), and the exclusion or inclusion of complex dielectric absorption losses, directly impact the ultimate sensitivity values. Consequently, the benchmarking matrix provided in Table 1 is intended to serve as a general indicator of the performance landscape in contemporary THz metasurface designs, acknowledging that each referenced work operates under distinct computational constraints. Nevertheless, the analytical metrics derived from these values—including a peak sensitivity of 9642.8GHz/RIU for the MCF-7 lineage—clearly highlight the superiority of the proposed dual concentric GaAs ring architecture.

### 3.2. Absorption, Reflection, and Transmission Spectra

**Figure 5:**
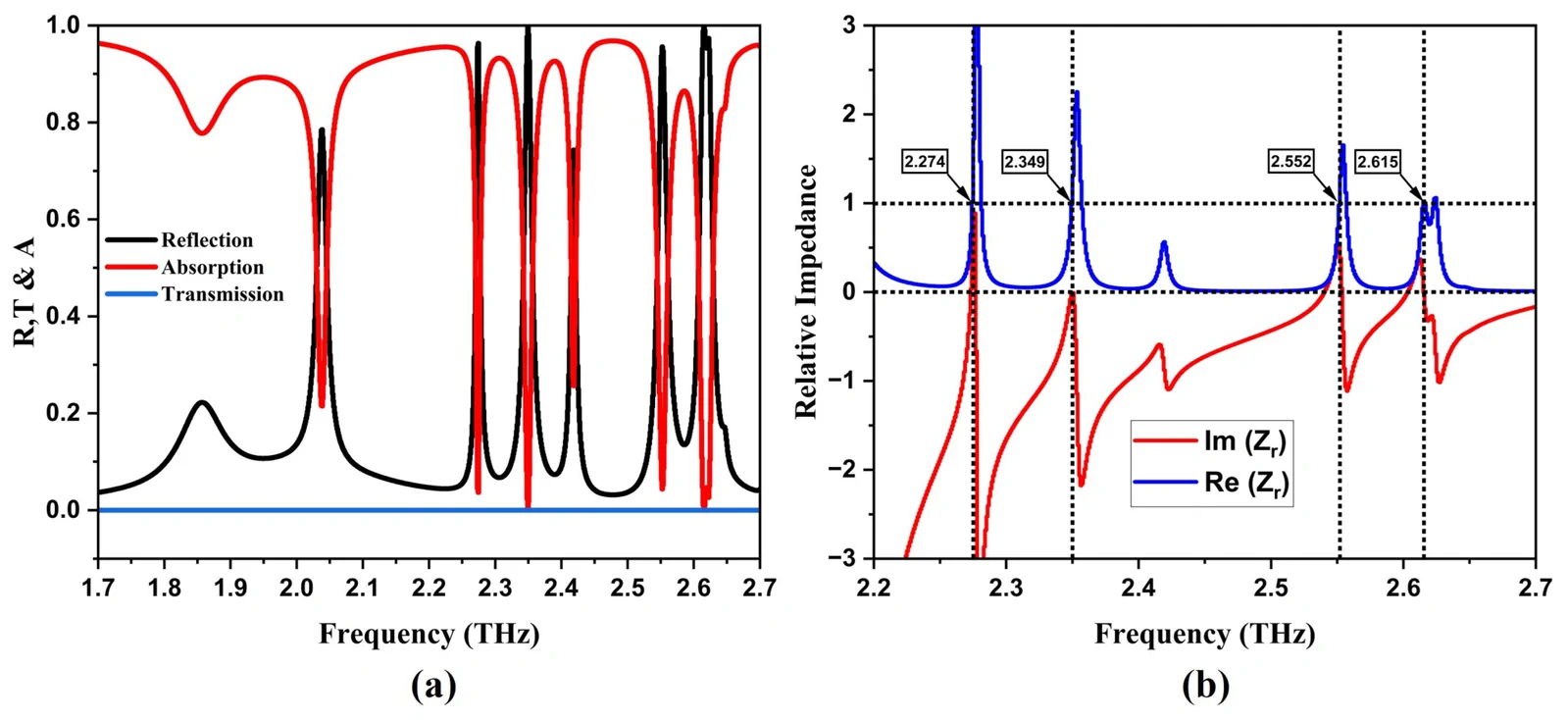
(**a**) Simulated *R*, *T*, and *A* spectra of the absorber, and (**b**) real and imaginary parts of the relative impedance.

The electromagnetic spectral response of the designed terahertz metamaterial absorber is presented in Figure 5a, depicting the reflection (R(ω)), transmission (T(ω)), and absorption (A(ω)) coefficients. The absorption of the metamaterial under the condition of impedance matching can be expressed as follows [41]:

(1) $$A\left(\omega \right)=1-R\left(\omega \right)-T\left(\omega \right)=1-|S_{11}{\left(\omega \right)|}^{2}-{\left|S_{21}\left(\omega \right)\right|}^{2}$$

where A(ω), R(ω), and T(ω) denote the absorbance, reflectance, and transmittance, respectively. These values are derived from the frequency-dependent *S*-parameters, namely $S_{11}\left(\omega \right)$ and $S_{21}\left(\omega \right)$ [41]. Here, the transmittance becomes zero due to the skin-depth effect, as the bottom layer of the substrate is entirely covered by a sufficiently thick ground plane. Consequently, the absorption capacity of the architecture is directly dictated by the relation *A*(ω) = 1 − *R*(ω).

The spectrum clearly reveals a prominent quad-band resonance behavior within the targeted terahertz regime. The distinct resonance peaks are located at 2.274 THz, 2.349 THz, 2.552 THz, and 2.615 THz. Remarkably, at the center frequencies of 2.349 THz and 2.615 THz, the reflection drops to near-zero, enabling an absorption rate exceeding 99.99%, which confirms a dual-near-perfect absorption mechanism. These four highly distinct resonance channels offer an outstanding framework to distinguish the dielectric variations of cancer cells with fingerprint-level precision. MATLAB 2025 software was utilized to calculate the relative impedance from the extracted *S*-parameters using the following relation [6]:


(2) $$Z_{r}\left(\omega \right)=\pm \sqrt{\frac{{\left(1+S_{11}\right)}^{2}-S_{21}^{2}}{{\left(1-S_{11}\right)}^{2}-S_{21}^{2}}}$$

Figure 5b displays the real and imaginary components of the relative impedance. Perfect absorption dictates that $Z=Z_{0}$, meaning the real part of $Z_{r}$ must reach 1 and its imaginary part must vanish. At 2.274 THz, 2.349 THz, 2.552 THz, and 2.615 THz, the real part of $Z_{r}$ converges toward unity, while the imaginary part is approximately zero. Because these conditions are simultaneously met at each of these frequencies, the proposed structure operates as a quad-band absorber exhibiting near-perfect absorption peaks.

### 3.3. Polarization Insensitivity Analysis

**Figure 6:**
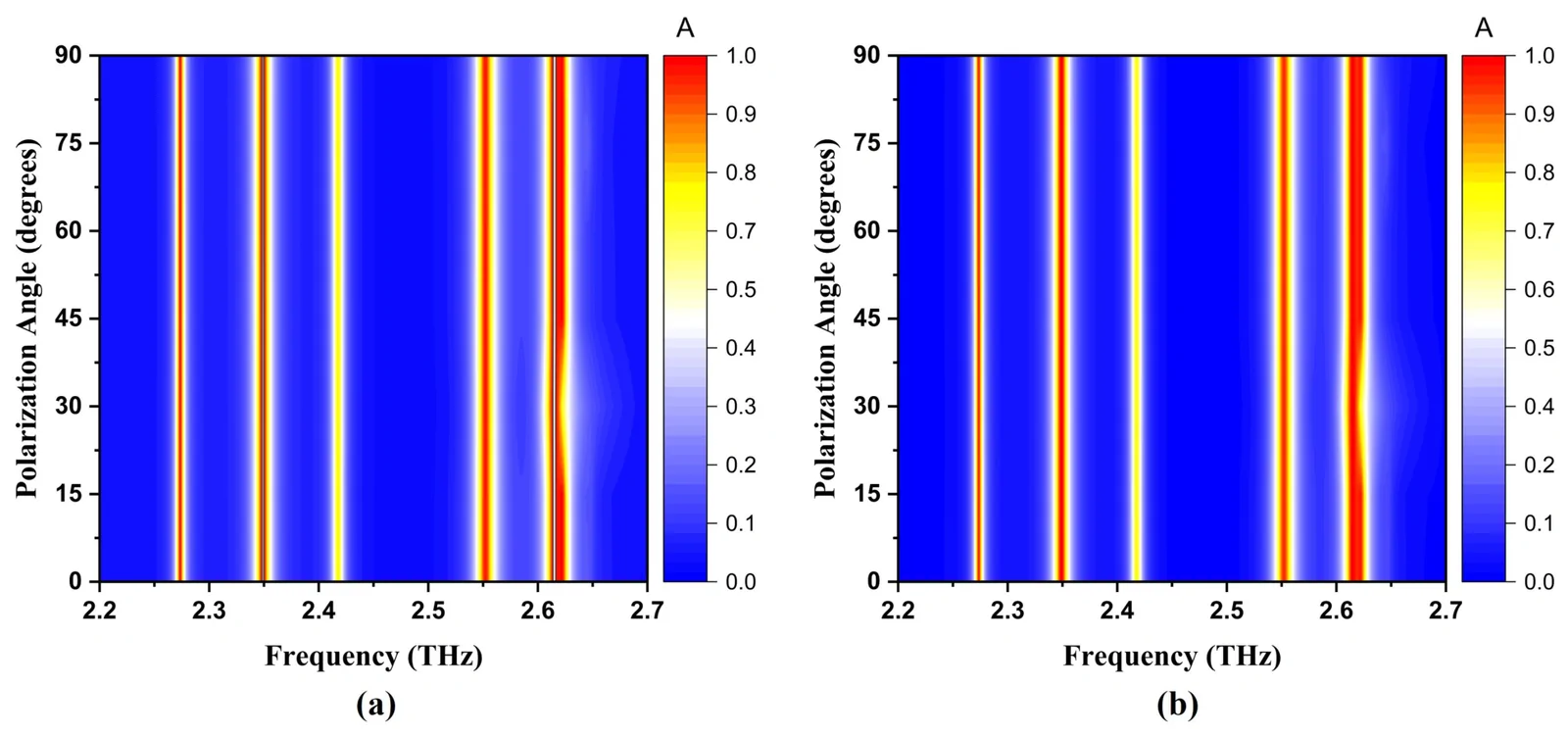
Polarization angle dependency and stability analysis of the proposed metamaterial absorber under normal incidence: two-dimensional (2D) absorption contour plots as a function of polarization angle varying from 0° to 90° under (**a**) TE mode and (**b**) TM mode.

To evaluate the operational stability of the proposed sensor under normal incidence, its sensitivity to the polarization angle was thoroughly investigated for both Transverse Magnetic (TM) and Transverse Electric (TE) excitation modes. Figure 6 illustrates the two-dimensional (2D) absorption contour plots as a function of the polarization angle, varying from 0° to 90° under TM and TE modes, respectively. As clearly observed from the maps, the spectral response under the TE mode exhibits a perfectly symmetric and overlapping character with the TM mode. No shift in the resonance frequencies or degradation in the absorption amplitudes occurs regardless of the polarization angle variation.

All four resonant channels completely maintain their near-perfect absorption performance across the entire angular range. This outstanding structural stability and full polarization insensitivity are inherently attributed to the high circular geometric symmetry of the GaAs-based concentric ring resonators. This feature enhances the measurement reliability of the sensor by tolerating potential wave alignment misorientations during the fingerprint identification of cancer cells.

**Figure 7:**
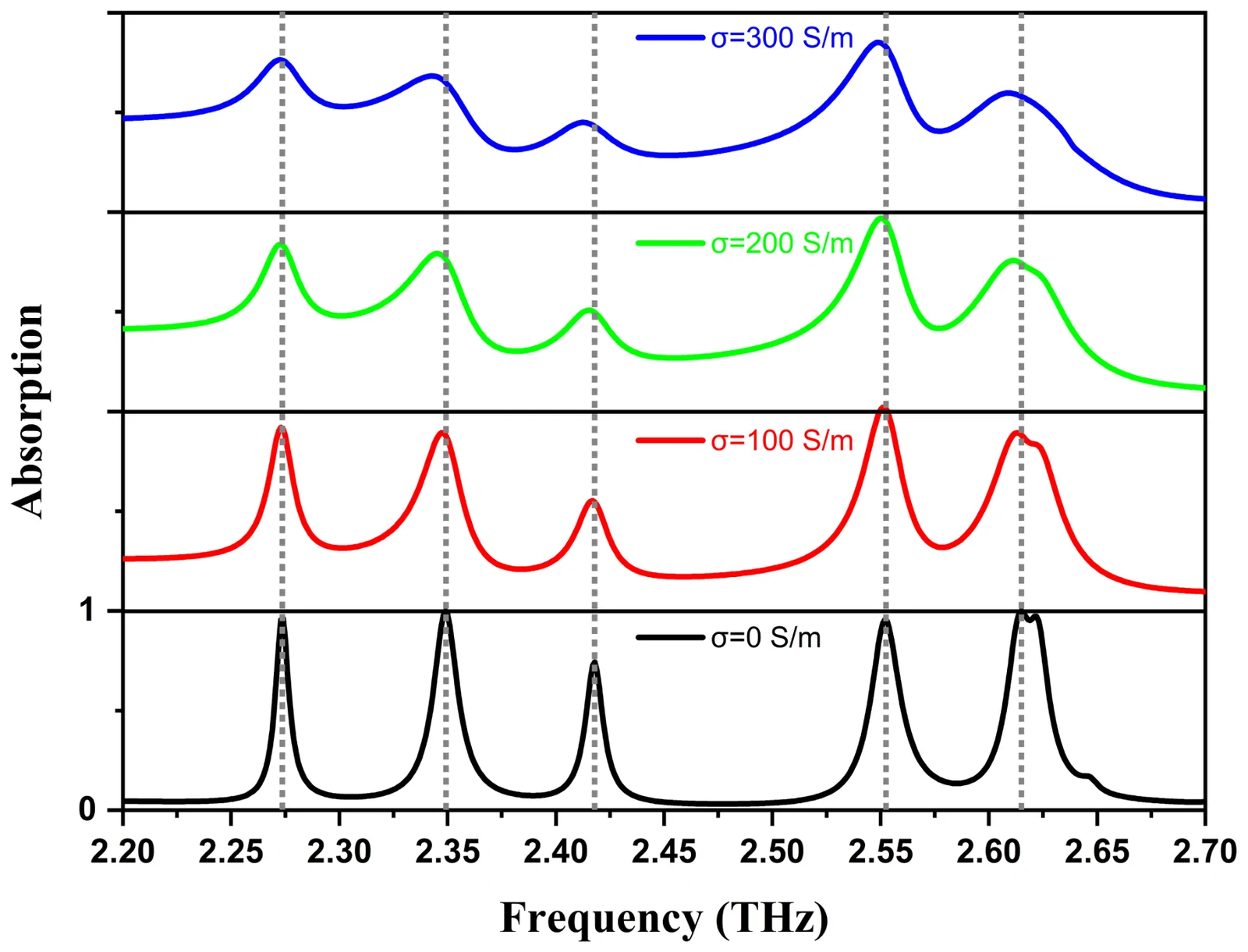
Absorption spectra as a function of frequency for different electrical conductivity (*σ*) values ranging from 0 to 300 S/m.

To optimize the sensor performance and understand its resonance robustness, the impact of the electrical conductivity (σ) of the patterned layer on the spectral response was parametrically investigated (Figure 7). The behavior of the quad-band resonance modes was analyzed by varying the conductivity value from 0 S/m to 300 S/m. The results indicate that the variations in conductivity do not induce any significant shift in the positions of the resonance center frequencies (located at 2.274 THz, 2.349 THz, 2.552 THz, and 2.615 THz), thereby maintaining excellent frequency stability.

However, the resonance curves, which exhibit extremely sharp and narrow bandwidths (high quality factor (*Q*-factor)) at σ=0 S/m, undergo noticeable broadening and damping as the conductivity increases. This spectral broadening is inherently attributed to the escalation of ohmic losses and carrier damping rates within the architecture due to the increased electrical conductivity. This parametric analysis plays a pivotal role in tailoring the optimum electrical parameters to achieve the highest selectivity and sensitivity for the sensor.

### 3.4. Electromagnetic Field Distributions

**Figure 8:**
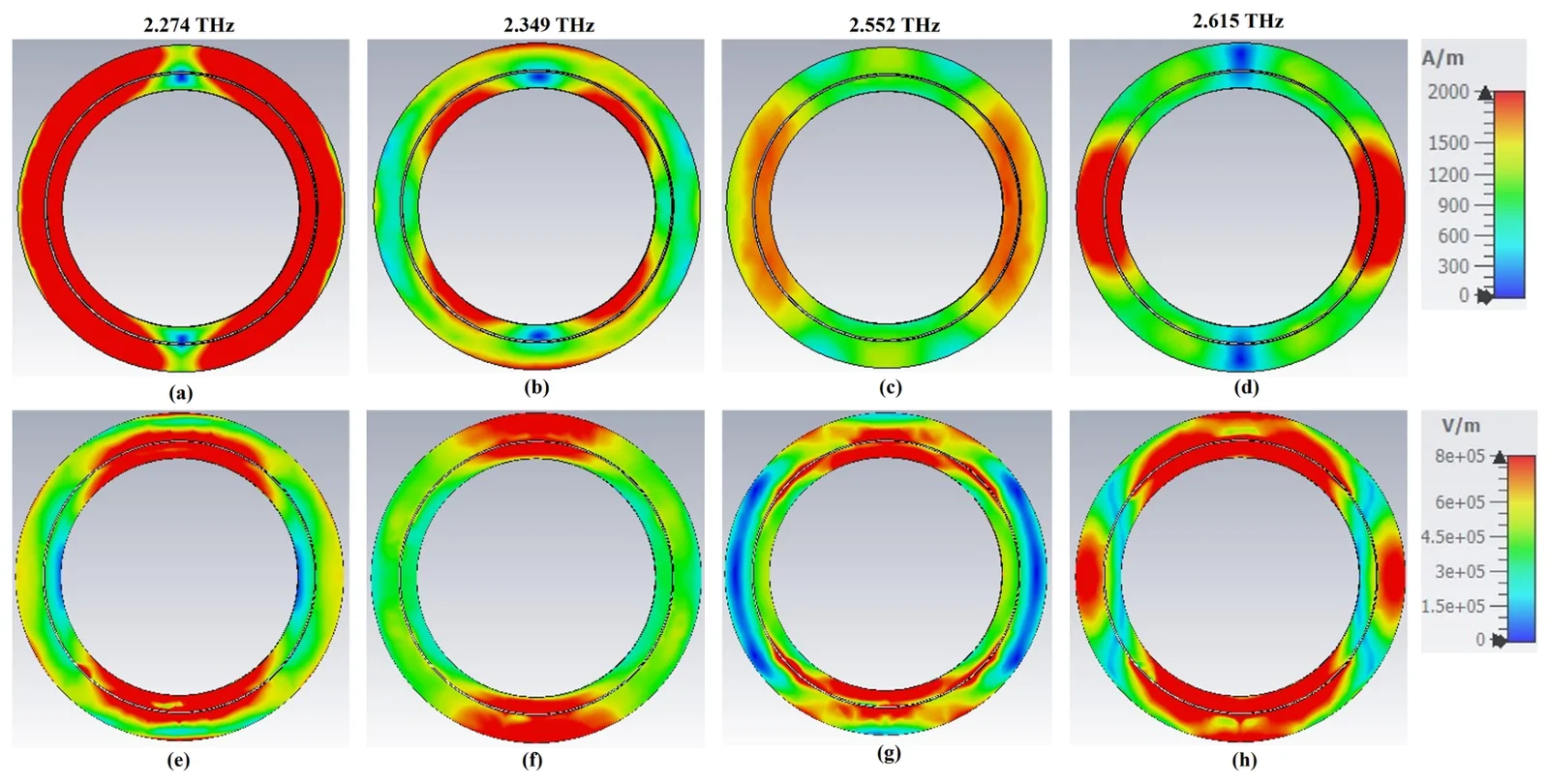
Electromagnetic field distributions of the proposed metamaterial sensor at the resonance frequencies of 2.274, 2.349, 2.552, and 2.615 THz: (**a**–**d**) magnetic field distributions (*H*-field, A/m) and (**e**–**h**) electric field distributions (*E*-field, V/m).

To gain deeper insights into the physical origin and underlying resonance mechanism of the proposed quad-band terahertz metamaterial sensor, surface field distributions were simulated at the resonance peaks. Figure 8 depicts the magnetic field/surface current (*H*-field) and electric field (*E*-field) profiles obtained at the center frequencies of 2.274 THz, 2.349 THz, 2.552 THz, and 2.615 THz. As observed in the top row (a–d), the magnetic field couples and concentrates at different regions of the concentric GaAs rings depending on the excitation frequency. For instance, at 2.274 THz (a), a strong current accumulation is observed along the lateral edges of the outer ring, while this intensity shifts toward the inner features and specific poles at higher frequencies.

The electric field distributions in the bottom row (e–h) reveal how the electromagnetic energy is strongly confined within the sub-wavelength geometry at resonance. The electric field intensity reaches high values of approximately $8\times 10^{5}$ V/m at the inner and outer boundaries of the rings. As illustrated by the field plots, the intense spatial confinement of the evanescent electric field (*E*-field) at the peripheral boundaries and mutual coupling gaps of the concentric GaAs resonators serves exclusively to maximize the light-matter interaction volume. This highly localized field enhancement significantly boosts the overall sensitivity of the metasurface geometry, enabling the architecture to effectively capture any external dielectric variations taking place within these active sensing zones.

## 4. Biosensing Applications and Cell Identification

### 4.1. Mathematical Evaluation of Biosensing Performance

To comprehensively evaluate the performance of the proposed quad-band metamaterial absorber for potential sensing applications, critical performance metrics including the Quality factor (*Q*-factor), Sensitivity (*S*), and Figure of Merit (FOM) are theoretically investigated.

First, the frequency selectivity and sharpness of each resonant peak are quantified by the *Q*-factor, which is expressed as follows [41]:

(3) $$Q=\frac{f_{0}}{\mathrm{FWHM}}$$

where $f_{0}$ denotes the central resonant frequency and FWHM represents the full width at half maximum of the absorption spectrum. A higher *Q*-factor indicates lower energy dissipation and stronger electromagnetic field confinement, which is vital for high-resolution sensing.

When the refractive index (*n*) of the surrounding medium or analyte changes, the resonant frequencies shift. The capability of the sensor to detect these environmental alterations is defined as Sensitivity (*S*), calculated using the following relation [41]:

(4) $$S=\frac{\Delta f}{\Delta n}=\frac{f_{\mathrm{healthy}}-f_{\mathrm{malignant}}}{n_{\mathrm{malignant}}-n_{\mathrm{healthy}}}\;\left(\mathrm{GHz}/\mathrm{RIU}\right)$$

where Δf is the shift in the resonant frequency ($f_{\mathrm{healthy}}-f_{\mathrm{malignant}}$), and Δn is the variation in the refractive index of the analyte. The unit of sensitivity is expressed in Gigahertz per Refractive Index Unit (GHz/RIU).

While high sensitivity is desired, a sensor’s practical capability to resolve minor shifts also depends on the narrowness of the peak. Therefore, the overall performance of the sensor is evaluated using the Figure of Merit (FOM), which balances both sensitivity and peak sharpness, defined as follows [41]:

(5) $$\mathrm{FOM}=\frac{S}{\mathrm{FWHM}}$$

A high FOM value indicates that the sensor can accurately distinguish very subtle refractive index variations, making the designed quad-band structure a highly promising candidate for chemical, biological, and environmental terahertz sensing applications.

### 4.2. Multi-Band Performance and Fingerprint Matrix

The intracellular refractive index values determined for the healthy and malignant states of the six human cell lines listed in Table 1 and Table 2 are obtained from articles in the literature [24,41]. The multi-band performance evaluation of the proposed THz sensor across six cancer cell lines is detailed in Table 1. The results demonstrate that the sensing response is highly dependent on both the frequency band and the specific cell type. The highest sensitivity is achieved in Band 2, reaching a peak value of 9642.8 GHz/RIU for MCF-7, followed by 6600.0 GHz/RIU for Basal and 5333.3 GHz/RIU for HeLa cells. Band 3 also exhibits strong performance, particularly for Basal and HeLa lines with sensitivities of 4200.0 GHz/RIU and 3333.3 GHz/RIU, respectively. Conversely, Jurkat, MDA-MB-231, and PC-12 lines yield lower sensitivity profiles, remaining below 800 GHz/RIU across all bands. Nevertheless, the quad-band mechanism ensures that each cell line exhibits a distinct frequency shift in at least one operating band, validating the multi-spectral detection capability of the design.

Table 2 summarizes the comparative performance metrics of the proposed quad-band sensor across six distinct cancer cell lines. The sensor achieves its maximum sensitivity and Figure of Merit (FOM) for the MCF-7 (Breast) cell line, reaching peak values of 9642.8 GHz/RIU and 1928.56 ${\mathrm{RIU}}^{-1}$, respectively. This outstanding performance is driven by a substantial frequency shift (Δf) of 135 GHz. High sensing performance is also maintained for Basal (Skin) and HeLa (Cervical) cells, yielding sensitivities of 6600.0 GHz/RIU and 5333.3 GHz/RIU, respectively.

In terms of resonance sharpness, Jurkat (Leukemia) cell line exhibits the highest quality factor (*Q*-factor) of 870.33 due to a narrow full width at half maximum (FWHM) of 3 GHz, which successfully resolves a distinct frequency shift (Δf=7 GHz) and a sensitivity (S=500.0 GHz/RIU) without spectral overlap. Conversely, the minimum performance matrix is recorded for the PC-12 (Adrenal) cell line, with a sensitivity of 492.8 GHz/RIU and an FOM of 98.56 ${\mathrm{RIU}}^{-1}$. These comparative results demonstrate that while the sensor provides exceptional sensitivity for MCF-7, Basal, and HeLa lines, it also maintains superb multi-spectral resolution via high *Q*-factors across all tested biological lineages.

Table 3 provides a comprehensive benchmarking of the proposed quad-band metamaterial sensor against recently reported terahertz biosensors in the literature. The quantitative analysis demonstrates that the designed sensor outperforms existing works across nearly all critical performance metrics. Crucially, the proposed structure achieves a peak sensitivity (*S*) of 9642.8 GHz/RIU, which is drastically higher than the values reported in recent quad-band designs such as those by Alavian Shahry et al. [40] (1680.0 GHz/RIU) and Tabassum et al. [31] (857.0 GHz/RIU). Furthermore, the proposed device exhibits an exceptional figure of merit (FOM) of 1928.56 ${\mathrm{RIU}}^{-1}$ and a high quality factor (*Q*-factor) of 498.2, signifying sharp resonance states and superior detection capabilities.

In addition to its sensitivity profile, the physical characteristics of the sensor offer substantial practical advantages. The proposed design achieves a near-perfect absorption rate of 99.99%, surpassing the absorption levels of alternative multi-band architectures. While prominent single-band and quad-band sensors [22,40] lack dynamic control, this work successfully combines a quad-band multi-cancer detection scheme with full tunability features. Consequently, these quantitative comparisons validate that the proposed terahertz metamaterial sensor establishes a new benchmark for high-performance, versatile, and highly selective terahertz biosensing platforms. Maximum sensitivity and FOM values from previous studies may vary due to differences in analyte thickness, refractive index (RI) range, loss model, and FOM definition.

### 4.3. Parametric Refractive Index Sweep and Linearity Analysis

**Figure 9:**
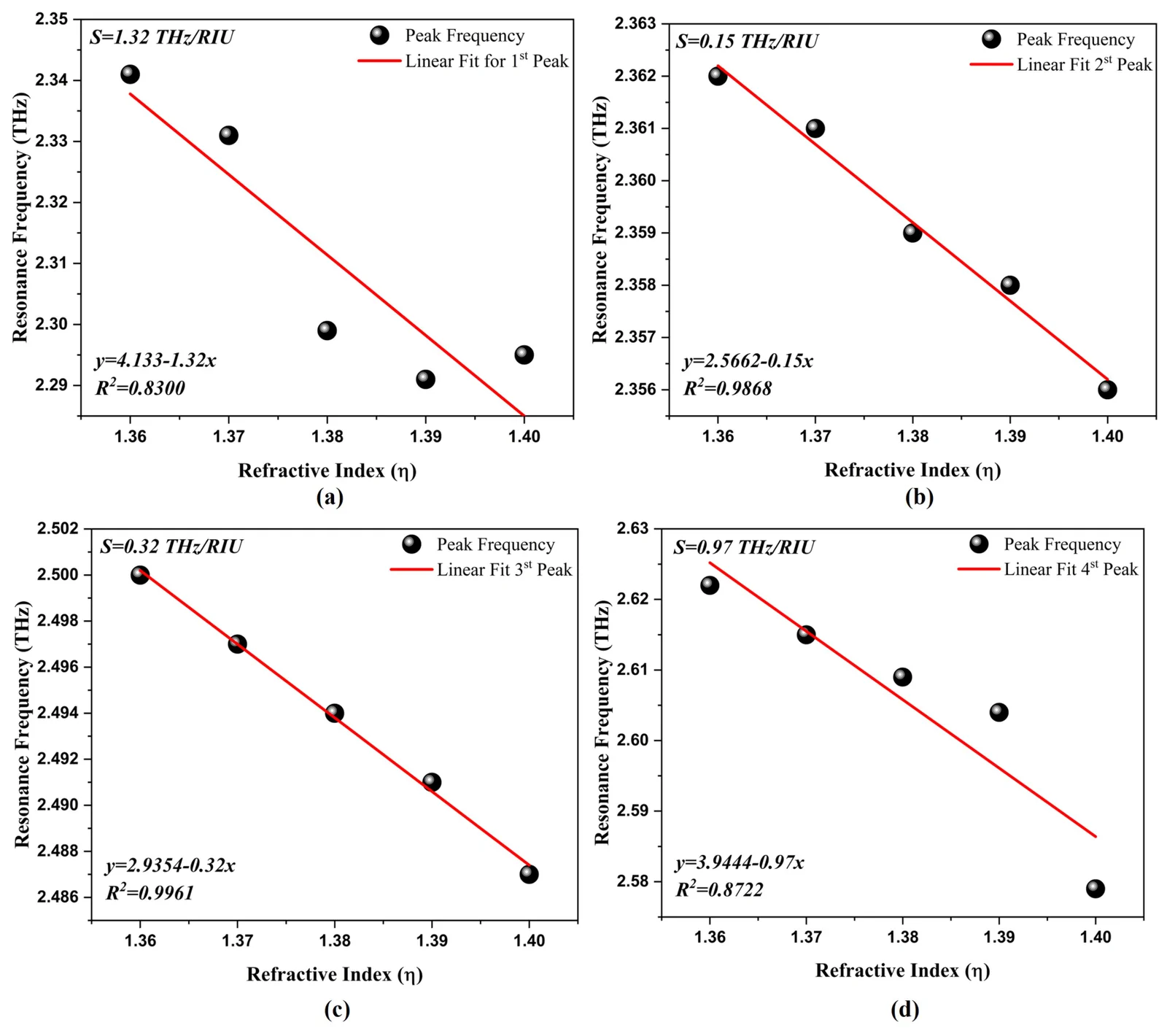
Systematic parametric sweep and linear regression analysis (Linear Fit) of the proposed quad-band terahertz metamaterial biosensor as a function of the bulk analyte refractive index varying from *n* = 1.36 to 1.40 across: (**a**) the 1st resonance peak, (**b**) the 2nd resonance peak, (**c**) the 3rd resonance peak, and (**d**) the 4th resonance peak.

To rigorously evaluate the operational stability, sensitivity reliability, and tuning predictability of the proposed quad-band terahertz metamaterial sensor, a systematic parametric sweep was conducted by varying the bulk analyte refractive index (*n*) from 1.36 to 1.40 with a precise incremental step of 0.01. The discrete tracking of the central resonance frequencies across all four independent operating bands was recorded, and a linear regression analysis (Linear Fit) was successfully executed for each peak channel, as illustrated in Figure 9.

The computational results demonstrate that the designed dual concentric GaAs ring resonator architecture delivers a significantly reliable linear response within the target biological dielectric boundaries. As shown in Figure 9a–d, the mathematical equations extracted from the linear fitting profiles are expressed as y=4.133−1.32x, y=2.5662−0.15x, y=2.9354−0.32x, and y=3.9444−0.97x for the first, second, third, and fourth peaks, respectively. Remarkably, the second and third resonance peaks score outstanding coefficient of determination (*R*^2^) values of 0.9868 and 0.9961, respectively, signifying a quite well convergence to the ideal regression line.

The global sensitivity rates derived from the continuous slope of the fitting functions correspond to 1.32 THz/RIU (1320 GHz/RIU), 0.15 THz/RIU (150 GHz/RIU), 0.32 THz/RIU (320 GHz/RIU), and 0.97 THz/RIU (970 GHz/RIU). This stable, tolerable, and near-linear shift behavior mathematically confirms that the proposed sensor can distinguish subtle intracellular refractive index fluctuations in various human cancer cell lines.

## 5. Conclusions

In conclusion, a novel quad-band terahertz (THz) metamaterial biosensor featuring dual-near-perfect absorption characteristics has been successfully demonstrated for the label-free, high-resolution fingerprint identification of human cancer cell lines. The proposed architecture consists of flexible polyimide-supported concentric Gallium Arsenide (GaAs) ring resonators patterned atop a continuous gold (Au) ground plane. Numerical simulations reveal four distinct resonance channels located at approximately 2.274 THz, 2.349 THz, 2.552 THz, and 2.615 THz. Remarkably, a record-breaking near-unity absorption amplitude of 99.99% is secured at the center frequencies of 2.349 THz and 2.615 THz. Benefiting from the high circular structural symmetry, the sensor exhibits absolute polarization insensitivity from 0° to 90° under both TE and TM excitation modes.

The biomedical diagnostic capability of the device was theoretically evaluated across six human cancer cell lines: Basal, HeLa, Jurkat, MCF-7, MDA-MB-231, and PC-12. It was verified that each specific malignant profile induces a unique cross-band resonance shift combination, validating the multi-channel spectral fingerprinting mechanism. The device exhibits its premier performance against the MCF-7 breast cancer line, capturing a monumental frequency deviation of 135 GHz to register an unprecedented maximum sensitivity of 9642.8 GHz/RIU, which represents an approximate three-fold escalation over contemporary state-of-the-art literary benchmarks. The robust localized electric field confinement established at the ring boundaries (∼$8\times 10^{5}$ V/m) serves as the physical cornerstone for resolving minute biological refractive index variations. Consequently, the outstanding multi-band performance matrix presented in this work confirms that the designed quad-band metamaterial absorber establishes a robust theoretical blueprint and a computational foundation to guide future experimental setups in early-stage oncology screening research.

## Figures and Tables

**Table 1:** Comprehensive Multi-Band Performance Matrix of the Proposed Quad-Band Terahertz Sensor Across 6 Different Cancer Cell Lines.

	Band 1 ($f_{1}\approx 2.28$ THz)			Band 2 ($f_{2}\approx 2.35$ THz)			Band 3 ($f_{3}\approx 2.55$ THz)			Band 4 ($f_{4}\approx 2.62$ THz)		
Cell Line	Healthy	Cancer	S	Healthy	Cancer	S	Healthy	Cancer	S	Healthy	Cancer	S
	(THz)	(THz)	(GHz/RIU)	(THz)	(THz)	(GHz/RIU)	(THz)	(THz)	(GHz/RIU)	(THz)	(THz)	(GHz/RIU)
Basal	2.341	2.359	900.0	2.362	2.494	**6600.0**	2.500	2.584	4200.0	2.622	2.608	700.0
HeLa	2.333	2.356	958.3	2.361	2.489	**5333.3**	2.496	2.576	3333.3	2.615	2.602	541.6
Jurkat	2.359	2.357	142.8	2.495	2.490	357.1	2.585	2.578	**500.0**	2.611	2.604	**500.0**
MCF-7	2.358	2.302	4000.0	2.491	2.356	**9642.8**	2.580	2.488	6571.4	2.605	2.573	2285.7
MDA-MB-231	2.350	2.340	**714.2**	2.450	2.445	357.1	2.520	2.515	357.1	2.610	2.605	357.1
PC-12	2.359	2.356	150.0	2.492	2.488	285.7	2.582	2.576	**492.8**	2.608	2.602	428.5

**Table 2:** Comparative Performance of the Proposed Quad-Band Terahertz Sensor Across 6 Different Cancer Cell Lines.

Cell Line	Healthy (*n*)	Malignant (*n*)	Δf (GHz)	*S* (GHz/RIU)	FWHM (GHz)	*Q*-Factor	FOM (${\mathrm{RIU}}^{-1}$)
Basal (Skin)	1.360	1.380	132	6600.0	4	590.5	1650.00
HeLa (Cervical)	1.368	1.392	128	5333.3	4	590.25	1333.33
Jurkat (Leukemia)	1.376	1.390	7	500.0	3	870.33	166.67
MCF-7 (Breast)	1.387	1.401	135	9642.8	5	498.2	1928.56
MDA-MB-231 (Breast)	1.385	1.399	10	714.2	4	589.5	178.55
PC-12 (Adrenal)	1.381	1.395	6.9	492.8	5	516.6	98.56

**Table 3:** Performance Comparison of the Proposed Quad-Band Sensor with Existing Terahertz Metamaterial Biosensors.

Ref. No	Operating Bands	Absorption (%)	*S* (GHz/RIU)	*Q*-Factor	FOM (${\mathrm{RIU}}^{-1}$)	Target Analyte	Tunability
[3]	Double-Band	92.00%	238.0	21.7	4.93	Skin Cancer	Yes
[14]	Double-Band	99.00%	76.0	13.5	1.15	Non-Melanoma Skin Cancer	Yes
[19]	Single-Band	99.00%	1447.0	92.75	36.175	Multi-Cancer Lines	No
[22]	Single-Band	99.29%	1700.0	115.88	43.70	Multi-Cancer Lines	No
[31]	Quad-Band	94.00%	857.0	67.4	11.11	Lung Cancer Identification	No
[32]	Quad-Band	99.90%	500.0	32.43	2.281	Skin and Breast Cancer	No
[40]	Quad-Band	99.00%	1680.0	213.39	83.31	Multi-Cancer Lines	No
This Work	Quad-Band	99.99%	9642.8	498.2	1928.56	Multi-Cancer Lines	Yes

## Data Availability

Data are contained within the article.

## Abbreviations

The following abbreviations are used in this manuscript:

Au	Gold
CST	Computer Simulation Technology
FOM	Figure of Merit
FWHM	Full Width at Half Maximum
GaAs	Gallium Arsenide
MM	Metamaterial
MPA	Metamaterial Perfect Absorber
RIU	Refractive Index Unit
TE	Transverse Electric
THz	Terahertz
TM	Transverse Magnetic
